# Abdominal fat distribution in endometrial cancer: from diagnosis to follow-up

**DOI:** 10.1186/s12885-025-14155-3

**Published:** 2025-05-15

**Authors:** Kristine E. Fasmer, Jostein Sæterstøl, Maria B. S. Ljunggren, Astrid M. K. Brun, Johanna M. A. Pijnenborg, Kathrine Woie, Camilla Krakstad, Ingfrid S. Haldorsen

**Affiliations:** 1 Mohn Medical Imaging and Visualization Centre (MMIV), Department of Radiology, Haukeland University Hospital, Bergen, Norway; 2 Section for Radiology, Department of Clinical Medicine, University of Bergen, Bergen, Norway; 3 Department of Obstetrics and Gynecology, Radboud university medical center, Nijmegen, The Netherlands; 4 Department of Obstetrics and Gynaecology, Haukeland University Hospital, Bergen, Norway; 5 Centre for Cancer Biomarkers, Department of Clinical Science, University of Bergen, Bergen, Norway

**Keywords:** Endometrial neoplasms, Computed tomography, Obesity, Adiposity, Intra-abdominal fat

## Abstract

**Background:**

The objective of this study is to quantify abdominal obesity markers from computed tomography (CT) scans at primary diagnosis and follow-up in a large endometrial cancer cohort, and to assess temporal change in obesity markers in relation to surgicopathological patient characteristics and outcome.

**Methods:**

Total- (TAV), subcutaneous- (SAV), visceral (VAV) abdominal fat volumes, and visceral-to-total fat percentage (VAV%) were derived from CT scans acquired in an endometrial cancer patient cohort at primary diagnosis (n_primary_=293). Temporal (delta, δ) changes in CT obesity markers from primary diagnosis to follow-up were assessed for all patients with a follow-up CT 13 (7, 19) [median (interquartile range)] months after diagnosis (n_follow−up_=152/293 patients). The CT obesity markers were assessed in relation to clinicopathological features and progression-free survival (PFS) using Mann-Whitney U-test, and Cox hazard ratios (HRs), respectively.

**Results:**

At primary diagnosis, VAV% was the only marker significantly associated with high-risk histology (median of 33% for endometrioid endometrial carcinoma (EEC) grade 1–2, 36% for EEC grade 3 and 36% for non-endometrioid EC, *p* = 0.003), myometrial invasion (MI) (median of 34% for MI < 50% vs. 35% for MI ≥ 50%, *p* = 0.03) and lymphovascular space invasion (LVSI) (median of 34% for no LVSI vs. 36% for LVSI, *p* = 0.009). High VAV% (≥ 35%) also predicted poor PFS both in univariable analysis (HR = 1.8, *p* = 0.02), and when stratified for surgicopathological FIGO stage (HR = 3.1, *p* = 0.03). At follow-up, median TAV, VAV, SAV, and VAV% were significantly lower than at primary diagnosis (*p* < 0.001 for all). Furthermore, patients with progression had larger reductions in visceral fat compartments (δVAV=-24%, δVAV% =-3%), than patients with no progression (δVAV=-17%, δVAV%=-2%, *p* ≤ 0.006 for both).

**Conclusion:**

Visceral abdominal obesity (high VAV%) is associated with high-risk histologic features, myometrial invasion, and poor prognosis. Furthermore, high visceral fat loss during/following therapy is associated with disease progression.

**Supplementary Information:**

The online version contains supplementary material available at 10.1186/s12885-025-14155-3.

## Introduction

Endometrial cancer is the sixth most common cancer in women worldwide, with 417,000 new cases in 2020 [1, 2]. Obesity is a well-established risk factor for endometrial cancer, with a reported > 50% higher cancer risk per each five-unit increase in body mass index (BMI) [3]. Obesity has also been linked to higher disease-specific- and all-cause mortality rates in endometrial cancer [4–6]. Although widely used, BMI is a rather crude and insufficient marker of obesity as it fails to discriminate fat- from muscle- and bone mass and does not distinguish visceral (VAV)- from subcutaneous abdominal fat volumes (SAV) [7].

Visceral fat located around the abdominal viscera in the mesentery and omentum, is known to exhibit distinctly different cellular-, molecular- and endocrine characteristics than that of subcutaneous fat [8]. Visceral obesity is associated with increased risk of cardiovascular disease, type 2 diabetes, and has also been linked to various cancers [9–11], and to more aggressive cancer phenotypes [12–16]. In endometrial cancer, computed tomography (CT)-assessed visceral obesity has been linked to poor survival in several studies (with *n* = 84–227 patients) [17–21]. However, to what extent abdominal fat distribution changes during and after primary endometrial cancer treatment, and whether change in fat distribution is associated with outcome, is not known.

In endometrial cancer care, abdominal CT is widely used at primary diagnostic work-up for diagnosing abdominal tumor spread. During follow-up, abdominal CT is commonly used for assessing treatment response, progression or recurrence after therapy [22, 23]. Although not routinely reported, visceral- and subcutaneous fat compartments can be segmented and quantified from abdominal CT scans [18, 24].

The present study presents CT-based abdominal fat distribution markers at primary diagnosis and during/after treatment in a large, population-based endometrial cancer cohort [25]. We further investigate whether fat distribution markers and change in these are associated with clinicopathological patient characteristics and patient outcome in endometrial cancer.

## Materials and methods

### Patient cohort and treatment

This retrospective study included 293 endometrial cancer patients treated at Haukeland University Hospital from 2016 to 2020. Haukeland University Hospital is a tertiary hospital for gynecologic oncology in the western regions of Norway, serving approximately 10% of the Norwegian population. The patients were all part of a larger prospectively collected population based endometrial cancer cohort (382 women in the study-time period) and were included in the present study if a preoperative CT examination of the abdomen/pelvis was available in our clinical picture archiving system (PACS) (*n* = 293/382 [77%] of the prospective cohort).

The patients were surgically staged according to FIGO (International Federation of Gynecology and Obstetrics) 2009 criteria, and clinicopathological- and follow-up data were collected from medical records (last accessed February, 2024). Median (interquartile range) [range] follow-up time for survivors was 47 (36, 59) [3, 79] months. Progression was defined as local recurrence or progression in the pelvis or new metastases in the abdomen or at distant sites.

Standard treatment was hysterectomy with bilateral salpingo-oophorectomy. Lymphadenectomy was performed in selected patients, based on preoperative risk assessments incorporating information on preoperative histology from curettage/endometrial biopsy and radiological findings [26–28]. Low-risk was defined as endometrioid endometrial carcinomas (EECs) grade 1–2 with < 50% myometrial invasion (MI) assessed by pelvic magnetic resonance imaging (MRI), intermediate-risk as EEC grade 1–2 with MI > 50% or EEC grade 3 with MI < 50%, and high-risk as EEC grade 3 with MI > 50% as well as all non-endometrioid endometrial carcinomas (NEECs). After primary surgical treatment with surgicopathological staging, high-risk patients were offered adjuvant treatment with standard chemotherapy (6 rounds of carboplatin and paclitaxel, at 3-week intervals [29]). The routine follow-up was clinical and gynecological examinations including vaginal ultrasound for all patients, and response evaluation with abdominal contrast enhanced (CE) CT for patients receiving adjuvant chemotherapy.

### CT imaging and analyses

Most of the included patients (n_primary_=293) had undergone preoperative CT as part of a combined positron emission tomography (PET)/CT examination (284/293 [97%]). Follow-up abdominal/pelvic CTs were available (in PACS) for 152/293 (52%) patients in n_primary_ (n_follow−up_=152) acquired at a median (interquartile range) [range] of 13 (7, 19) [2, 41] months after primary diagnosis. Of these, 104/152 (68%) were referred to follow-up CTs for routine evaluation of chemotherapy response or investigation of progressing/recurrent disease, 17/152 (11%) were referred for routine CT controls (no clinical symptoms), and 19/152 (13%) were referred for investigation of other symptoms/diseases. For the remaining 12/152 (8%) patients, the CT referrals were unavailable in our PACS (images imported from other institutions).

At preoperative imaging, 225/293 (77%) of the examinations were performed as low-dose CTs with no CT contrast agent (used primarily for attenuation correction of PET images), while 68/293 (23%) were CE CTs with diagnostic image quality. At follow-up, 5/152 (3%) of the examinations were performed as low-dose non-CE CTs, 7/152 (5%) were diagnostic non-CE CTs, while the remaining 140/152 (92%) were diagnostic CE CTs.

**Fig. 1:**
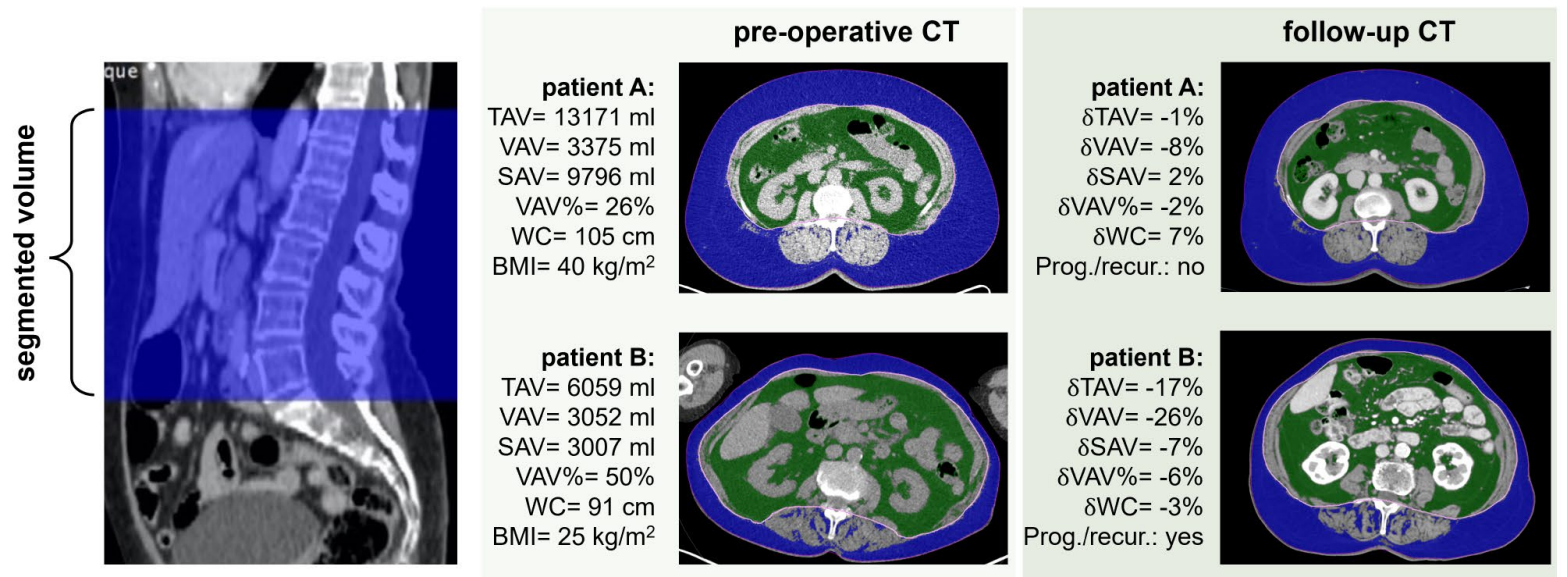
*Left:* Abdominal fat compartments are derived from computed tomography (CT) scans covering the level of the upper right diaphragm to the level of vertebra L5/S1 are segmented using the software iNtuition (TeraReon Inc San Mateo, USA) *Middle*: CT obesity markers derived at primary diagnosis for patient A (63 years, FIGO IIIB, EEC grade 2, BMI 40 kg/m^2^, alive and well at follow-up) and patient B (86 years, FIGO II, NEEC, BMI 25 kg/m^2^, recurrence detected 6 months after primary treatment) *Right*: Temporal (delta, δ) changes in CT obesity markers from primary treatment to follow-up for patient A and B BMI, body mass index, EEC, endometrioid endometrial carcinoma; NEEC, non-endometrioid endometrial carcinoma; FIGO, international federation of gynecology and obstetrics; SAV, subcutaneous abdominal fat volume; TAV, total abdominal fat volume; VAV, visceral abdominal fat volume; VAV%, visceral-to-total abdominal fat percentage; WC, waist circumference at L3/L4

Abdominal fat compartments, from the level of upper right diaphragm to the level of vertebra L5/S1, were segmented using the software iNtuition (TeraRecon Inc., San Mateo, USA) (Fig. 1). The segmentations were performed semi-automatically on axial CT images, with Hounsfield units (HU) in the range of -195 to -45, classified as adipose tissue [18, 20, 24]. If needed, the segmented visceral- and subcutaneous compartments were manually adjusted. Breast tissue was excluded from subcutaneous compartment if present in the selected CT field of view. The CT morphometric obesity markers; total abdominal fat volume (TAV, ml), subcutaneous abdominal fat volume (SAV, ml), visceral abdominal fat volume (VAV, ml), visceral-to-total fat percentage (VAV%), and waist circumference (WC, cm) measured at the level of L3/L4, were derived for all patients in the cohort at primary diagnosis (n_primary_=293) by reader A (MBSL) and for patients with a follow-up CT (n_follow−up_=152) by reader B (JS) (Fig. 1; Table 1). Additionally, both readers segmented 21 overlapping cases, for assessment of interreader variability for the CT obesity markers.

**Table 1:** Clinicopathological characteristics of endometrial cancer patients with computed tomography (CT) at primary diagnosis (n_primary_=293) and with CT at follow-up (n_follow−up_=152)

	*n*_primary_	*n*_follow−up_	p*
Age (years), median [range]	69 [27, 90]	70 [27, 90]	0.14
BMI (kg/m^2^), median [range]	28 [17, 59]	28 [17, 57]	0.22
Diabetes^a^, n (%)			0.48
no	256 (88)	130 (86)	
yes	36 (12)	21 (14)	
Menopausal status^a^, n (%)			0.16
pre/perimenopausal	24 (8)	8 (5)	
postmenopausal	267 (92)	143 (95)	
Preoperative risk^a^, n (%)			**< 0.001**
low-risk (EEC grade 1–2)	175 (62)	62 (42)	
high-risk (EEC grade 3/NEEC)	106 (38)	85 (58)	
Primary treatment, n (%)			0.75
hysterectomy	283 (97)	146 (96)	
curettage/palliative surgery	10 (3)	6 (4)	
Lymphadenectomy, n (%)			**< 0.001**
pelvic	86 (29)	47 (31)	
pelvic and paraaortic	70 (24)	53 (35)	
not performed	137 (47)	52 (34)	
Lymph node metastasis^b^, n (%)			**0.02**
no	130 (83)	78 (78)	
yes	26 (17)	22 (22)	
Myometrial invasion^a, b^, n (%)			0.12
< 50%	149 (53)	70 (48)	
≥ 50%	133 (47)	75 (52)	
Lymphovascular space invasion^a, b^, n(%)			0.07
no	215 (80)	103 (75)	
yes	54 (20)	34 (25)	
Histological subtype/grade^b^, n (%)			**< 0.001**
EEC grade 1–2	186 (63)	64 (42)	
EEC grade 3	32 (11)	26 (17)	
NEEC	75 (26)	62 (41)	
FIGO stage^b^, n (%)			**< 0.001**
I	213 (73)	86 (57)	
II	26 (9)	19 (12)	
III	35 (12)	32 (21)	
IV	19 (6)	15 (10)	
Additional treatment, n (%)			**< 0.001**
no	169 (58)	44 (29)	
chemotherapy	113 (39)	102 (67)	
radiotherapy	4 (1)	4 (2)	
chemoradiotherapy	3 (1)	1 (1)	
hormonal treatment	2 (1)	1 (1)	
other	2 (1)	-	
Progressing or recurrent disease			**< 0.001**
no	227 (77)	99 (65)	
yes	66 (23)	53 (35)	

BMI, body mass index; EEC, endometrioid endometrial carcinoma; FIGO, International Federation of Gynecology and Obstetrics; NEEC, non-endometrioid endometrial carcinoma

^a^Number (n) of patients with variables missing/not assessed in n_primary_: BMI (8), diabetes (1), menopausal status (2), preoperative risk (12), myometrial invasion (11), lymphovascular space invasion (24)

^b^Surgicopathological assessments

*Mann-Whitney U-test for continuous variables (exact p), and Fisher’s exact test for categorical variables, for comparing patients with a follow-up CT (n_follow−up_=152) with patients without follow-up CT (*n* = 141). *p* < 0.05 marked in bold

### Statistical analyses

Interobserver reproducibility was assessed by intraclass correlation coefficients (ICCs) with two-way random-effects model (absolute agreement). Correlations between the CT obesity markers, age, and body mass index (BMI) at primary diagnosis were analyzed by Spearman’s rank correlation (rho [*ρ*]). The CT obesity markers at primary diagnosis, were assessed in relation to the clinicopathological features: age, diabetes, pathologically verified tumor histology/grade, myometrial invasion (MI), lymphovascular invasion (LVSI), lymph node metastases (LNM), and FIGO stage, using Mann-Whitney U test.

The obesity markers were dichotomized at median and univariable Cox Proportional Hazards Regression analyses were used to assess the obesity markers for prediction of progression-free survival (PFS). Obesity markers with significant univariable hazard ratios (HRs) were further stratified for FIGO stage, and interaction terms for significant covariable features were added. Kaplan-Meier plots with Mantel-Cox log-rank test were applied to depict PFS for patients grouped into quartiles for the obesity markers.

Temporal (delta [δ]) changes in CT obesity markers from primary diagnosis to follow-up were assessed in n_follow−up_, using Wilcoxon matched-pairs signed rank test. The delta CT obesity markers were further assessed in relation to clinicopathological features and disease progression using Mann-Whitney U test. As for the primary CT obesity markers, Cox Proportional Hazards Regression analyses and Kaplan Meier plot with Mantel-Cox log-rank test were used to assess selected delta obesity markers for prediction of PFS.

All statistical analyses were performed with Stata 17.0 (StataCorp., College Station. TX, USA). The *p*-values were two-sided, exact and considered statistically significant if < 0.05.

## Results

### Clinicopathological patient characteristics

Clinicopathological characteristics are given in Table 1 for the 293 patients with CT at primary diagnosis (n_primary_), and for the subcohort of 152 patients having an eligible CT follow-up (n_follow−up_). A larger proportion of patients in n_follow−up_ had high-risk histology (EEC grade 3: 17%; NEEC: 41%), advanced FIGO stage (FIGO II-IV: 43%) and underwent adjuvant treatment (71%) compared to patients in n_primary_ (EEC grade 3: 11%; NEEC: 26%, FIGO II-IV: 27% and adjuvant treatment: 43%) (Table 1). BMI was available for 285/293 of the patients at primary diagnosis (Table 1), but patient weight/BMI was not systematically measured/recorded in the medical records at time of CT follow-up.

### Patient obesity markers at primary diagnosis

#### Correlation between CT obesity markers, BMI, and patient age

At primary diagnosis, the patients had a median BMI of 28 kg/m^2^ and median age of 69 years (Table 1). Interobserver reproducibility for all extracted CT obesity markers was excellent with ICCs ≥ 0.94 (Suppl. Table S1). TAV, VAV, SAV and WC were all highly correlated to BMI (*ρ* ≥ 0.78, *p* < 0.001 for all), while VAV% was not (*ρ* = -0.12, *p* = 0.05) (Suppl. Table S2). VAV% was moderately correlated to age (*ρ* = 0.41, *p* < 0.001), while the remaining CT derived obesity markers had a weak (SAV *ρ* = -0.17, *p* < 0.05) or no correlation to age (TAV, VAV, WC: -0.09 ≤ *ρ* ≤ 0.06, *p* > 0.05 for all). TAV, VAV, SAV and WC were all highly correlated to each other (*ρ* ≥ 0.79, *p* < 0.001 for all), while VAV% was only weakly correlated to VAV (*ρ* = 0.35, *p* < 0.001) and SAV (*ρ* = -0.22, *p* < 0.001) (Suppl. Table S2).

#### Obesity markers in relation to clinicopathological patient characteristics

Among the assessed CT obesity markers, VAV% was the only marker significantly associated with both high-risk histology (median VAV% of 33% for EEC grade 1–2, 36% for EEC grade 3, and 36% for NEEC; *p* = 0.003), myometrial invasion (median VAV% of 34% for MI < 50% and 35% for MI ≥ 50%; *p* = 0.03), and lymphovascular space invasion (median VAV% of 34% for no LVSI and 36% for LVSI; *p* = 0.009) (Table 2). Higher VAV% was also seen in patient with higher age (≥ 69 years) and with diabetes (*p* ≤ 0.002 for both, Table 2).

**Table 2:** Computed tomography (CT) derived abdominal obesity markers and body mass index (BMI) in relation to clinicopathological features at primary diagnosis in n_primary_=293 endometrial cancer patients

	*n* (%)	TAV (ml) median [95% CI]	p*	VAV (ml) median [95% CI]	p*	SAV (ml) median [95% CI]	p*	VAV% median [95% CI]	p*	WC (cm) median [95% CI]	p*	BMI^a^ (kg/m^2^) median [95% CI]	p*
Age			0.15		0.53		**0.01**		**< 0.001**		0.71		0.29
< 69 years	146	9755 [8195, 11233]		3100 [2788, 3394]		6713 [5628, 7256]		33 [30, 34]		99 [95, 103]		28 [27, 30]	
≥ 69 years	147	8423 [7961, 9125]		3250 [3054, 3446]		5201 [4857, 5666]		36 [35, 37]		97 [95, 100]		28 [27, 28]	
Diabetes^a^			**0.02**		**< 0.001**		0.13		**0.002**		**0.002**		0.08
no	256	8524 [8117, 9333]		3100 [2908, 3259]		5534 [5194, 6069]		34 [33, 35]		97 [95, 99]		28 [27, 29]	
yes	36	10,507 [8501, 12106]		4050 [3354, 4948]		6475 [5200, 7406]		37 [34, 41]		103 [99, 109]		28 [27, 32]	
Lymph node metastases^b^			0.47		0.85		0.29		0.51		0.24		0.63
no	130	8893 [8172, 10003]		3210 [2854, 3354]		5894 [5220, 6544]		34 [33, 35]		97 [95, 101]		28 [27, 29]	
yes	26	8591 [5838, 10459]		3437 [2309, 4031]		5228 [3536, 6774]		35 [32, 38]		96 [83, 102]		28 [23, 30]	
Myometrial invasion^a^			0.92		0.42		0.53		**0.03**		0.71		0.98
< 50%	149	8896 [7959, 9877]		3083 [2757, 3272]		5892 [5281, 6592]		34 [32, 35]		97 [95, 100]		28 [27, 29]	
≥ 50%	133	8788 [8123, 9668]		3276 [3058, 3508]		5494 [5056, 6580]		35 [34, 36]		99 [96, 101]		28 [27, 29]	
Lymphovascular space invasion^a^			0.36		0.99		0.11		**0.009**		0.34		0.24
no	215	8946 [8200, 9912]		3237 [3017, 3337]		5892 [5479, 6613]		34 [33, 35]		98 [96, 101]		28 [28, 29]	
yes	54	8677 [7865, 9534]		3132 [2628, 3570]		5253 [4449, 6002]		36 [34, 38]		96 [94, 108]		27 [25, 29]	
Histological subtype/grade			0.08		0.21		**0.02**		**0.003**		0.11		**0.02**
EEC grade 1–2	186	9055 [8153, 10254]		3220 [3028, 3314]		6290 [5368, 6839]		33 [32, 35]		99 [97, 102]		28 [28, 30]	
EEC grade 3	32	8304 [5454, 10028]		2829 [2022, 3572]		5079 [3605, 6312]		36 [31, 38]		98 [91, 101]		27 [24, 9]	
NEEC	75	8482 [7730, 9529]		3449 [2904, 3688]		5296 [4574, 5930]		36 [35, 38]		96 [91, 101]		27 [25, 28]	
FIGO stage			0.35		1.00		0.15		0.23		0.47		0.47
I-II	239	8946 [247, 9497]		3185 [3022, 3310]		5855 [5294, 6422]		35 [33, 35]		98 [96, 100]		28 [27, 29]	
III-IV	54	8438 [7592, 10380]		3400 [2491, 3970]		5221 [4576, 6501]		36 [33, 37]		97 [93, 102]		28 [26, 30]	

CI, confidence interval; EEC, endometrioid endometrial carcinoma; FIGO, International Federation of Gynecology and Obstetrics; NEEC, non-endometrioid endometrial carcinoma; SAV, subcutaneous abdominal fat volume; TAV, total abdominal fat volume; VAV, visceral abdominal fat volume; VAV%, visceral-to-total fat percentage; WC, waist circumference

^a^Number (n) of patients with variables missing/not assessed in n_primary_: BMI (8), diabetes (1), myometrial invasion (11), lymphovascular space invasion (24)

^b^Lymphadenectomy in 156/293 of the patients in n_primary_

*Mann-Whitney U-test for two categories (exact p). Kruskal-Wallis equality-of-populations rank test for three categories. *p* < 0.05 marked in bold

Similar to the VAV%, TAV, VAV and WC were all significantly higher in patients with diabetes (*p* ≤ 0.02), but none of these obesity markers were associated with MI or LVSI, *p* ≥ 0.11 for all). SAV was significantly lower in high-risk histology (*p* = 0.02) and TAV tended to the same (*p* = 0.08) (Table 2). None of the assessed obesity markers were significantly linked to presence of LNM (*p* ≥ 0.24 for all) or to FIGO stage (*p* ≥ 0.15 for all) (Table 2).

#### Obesity markers and progression-free survival

In univariable Cox regression analyses, none of the obesity markers were significantly associated with PFS when analyzed as continuous variables (results not shown). However, when dichotomized at median, VAV% ≥35% predicted poor PFS with a HR of 2.3 (*p* = 0.03), while neither TAV, VAV, SAV, WC nor BMI were significantly associated with PFS (*p* ≥ 0.24 for all) (Suppl. Table S3). To account for the (moderate) positive correlation between VAV% and patient age (*ρ* = 0.41, Suppl. Table S2), an interaction term between VAV% and age was incorporated (*p* = 0.23), and the covariance of VAV% and age in the Cox model was assessed (correlation: -0.27). VAV% ≥35% also predicted PFS when stratified for FIGO stage I-IV (HR = 3.1, *p* = 0.005) (Suppl. Table S3).

**Fig. 2:**
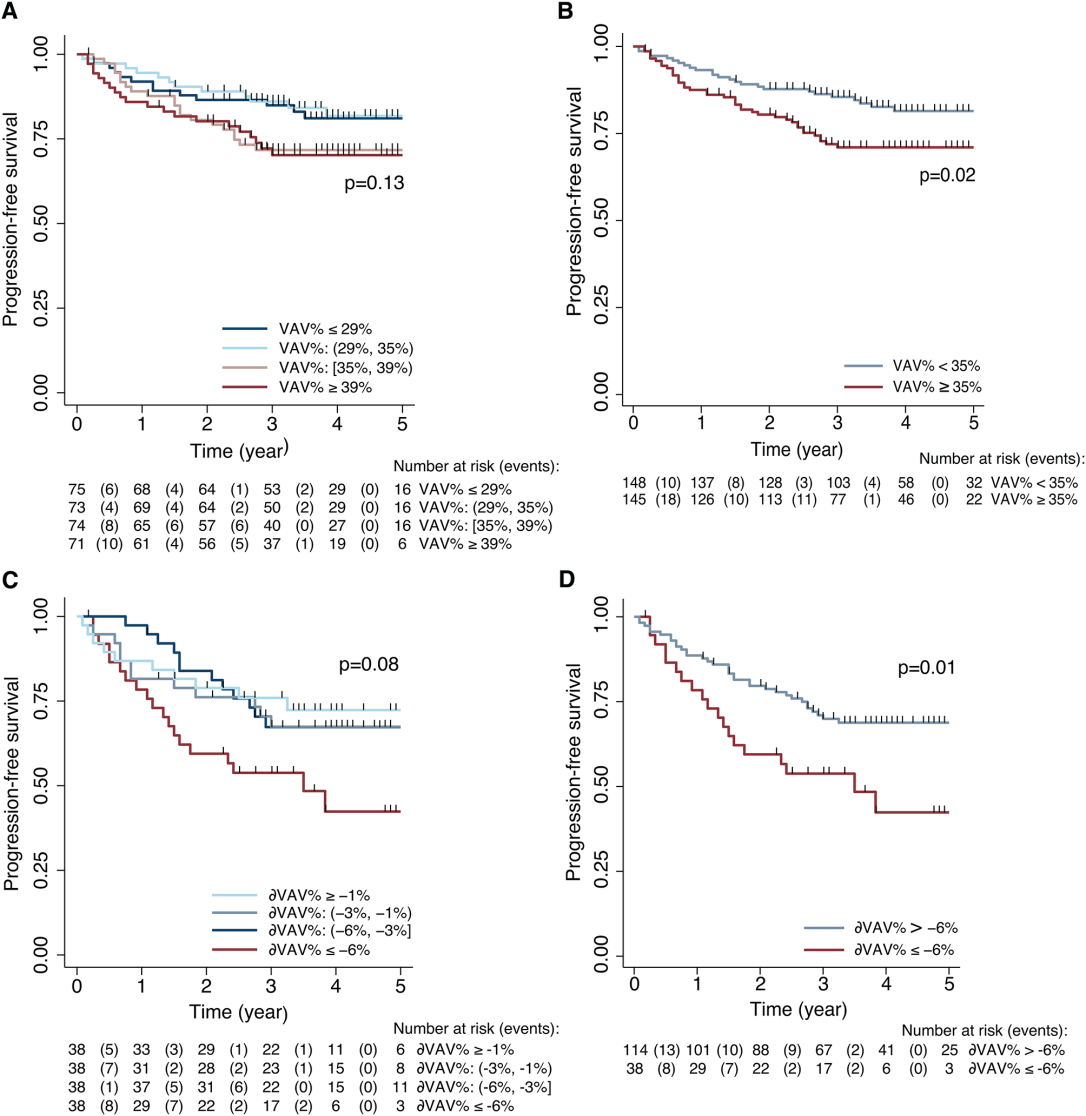
Kaplan-Meier curves depicting VAV% (**A**) and delta [δ]VAV% (**C**) grouped in quartiles in relation to progression-free survival (PSF). Patients with VAV%≥35% (**B**) and δVAV%≤-6% (**D**) have significantly reduced PFS compared to patients with VAV%<35%, and δVAV%>-6%, (*p* ≤ 0.02 for both)

Kaplan-Meier survival curves for patients grouped in VAV% quartiles (quartile 1–4, Fig. 2A), show significantly lower PFS in patients with VAV% ≥35% (median) than in patients with VAV%<35% (median) (*p* = 0.02, Fig. 2B).

### Patient obesity markers at follow-up

#### Temporal changes in CT obesity markers from primary treatment to follow-up

At CT follow-up, median TAV, VAV, SAV, and VAV% were all significantly lower than at primary diagnosis (δTAV [-13%], δVAV [-20%], δSAV [-9%], δVAV% [-3%]; *p* < 0.001 for all), while no significant difference was observed for WC (*p* = 0.18) (Table 3). All delta CT obesity markers (δTAV, δVAV, δSAV and δWC%) showed a greater reduction in patients aged ≥ 69 years compared to those aged < 69 years (*p* ≤ 0.01 for all), except for δVAV%, which was similar (-3%) in both age groups (*p* = 0.41). Furthermore, more negative δVAV and δVAV% were observed in patients with high-risk histology (*p* ≤ 0.04 for both). LVSI was also associated with a larger negative δVAV (*p* = 0.004). For the remaining clinicopathological features (diabetes, LNM, MI, FIGO) there were no significant differences in the delta obesity markers (Table 4).

**Table 3:** Change (delta [δ]^a,b^, %) in computed tomography (CT)-derived abdominal obesity markers from primary diagnosis to CT follow-up scans 13 (7, 19) [median (interquartile range)] months after primary diagnosis, in all (n_follow−up_=152); and in patients developing (*n* = 53)/not developing (*n* = 99) progression/recurrence. Follow-up time for patients with no progression/recurrence (*n* = 99) was 47 (36, 60) [median (interquartile range)] months. For patients developing progression/recurrence (*n* = 53) time from primary diagnosis to progression/recurrence was 16 (8, 27) [median (interquartile range)] months

	All (*n = 152)* Median [95% CI]	p*	Progression/recurrence		
			Yes (*n = 53)* Median [95% CI]	No (*n = 99)* Median [95% CI]	p**
δTAV^a^	-13% [-16, -10]	**< 0.001**	-17% [-23, -12]	-11% [-14, -8]	**0.03**
δVAV^a^	-20% [-23, -17]	**< 0.001**	-24% [-30, -19]	-17% [-21, -13]	**0.006**
δSAV^a^	-9% [-12, -7]	**< 0.001**	-13% [-22, -7]	-8% [-11, -6]	0.27
δVAV%^b^	-3% [-4, -2]	**< 0.001**	-3% [-5, -3]	-2% [-3, -1]	**0.003**
δWC^a^	1% [0, 3]	0.18	1% [-3, 4]	1% [0, 3]	0.71

CI, confidence interval; δ, delta ((follow-up – primary)/primary); SAV, subcutaneous abdominal fat volume; TAV, total abdominal fat volume; VAV, visceral abdominal fat volume; VAV%. visceral-to-total fat percentage; WC, waist circumference

^a^Delta[δ] TAV, VAV, SAV and WC defined as ((follow-up - primary)/primary))

^b^Delta[δ] VAV% defined as (follow-up - primary)

*Wilcoxon matched-pair signed rank test (*p* < 0.05 marked in bold)

**Mann-Whitney U test exact p (*p* < 0.05 marked in bold)

**Table 4:** Change (delta [δ]^a,b^, %) in computed tomography (CT) derived abdominal obesity markers in relation to clinicopathological features at primary diagnosis in n_follow−up_=152 endometrial cancer patients

	n (%)	δTAV^a^ median [95% CI]	p*	δVAV^a^ median [95% CI]	p*	δSAV^a^ median [95% CI]	p*	δVAV%^b^ median [95% CI]	p*	δWC^a^ median [95% CI]	p*
Age			**0.004**		**0.01**		**0.009**		0.41		**< 0.001**
< 69 years	67	-9% [-13, -3]		-17% [-20, -12]		-7% [-10, -1]		-3% [-3, -1]		4% [1, 5]	
≥ 69 years	85	-16% [-19, -12]		-23% [-27, -19]		-11% [-16, -8]		-3% [-4, -2]		-1% [-2, 1]	
Diabetes^c^			0.25		0.58		0.23		0.49		0.09
No	130	-13% [-16, 10]		-19% [-23, -16]		-9% [-12, -6]		-3 [-3, -2]		2% [0, 3]	
Yes	21	-17% [-27, -7]		-22% [-31, -14]		-16% [-24, -4]		-3 [-4, -1]		-3% [-5, 3]	
Lymph node metastases^d^			0.90		0.58		0.69		0.36		0.99
no	78	-13% [-16, -10]		-19% [-24, -12]		-9% [-13, -7]		-2% [-3, -1]		1% [0, 4]	
yes	22	-11% [-20, -4]		-21% [-30, -16]		-10% [-16, 6]		-4% [-5, -2]		2% [-1, 3]	
Myometrial invasion^c^			0.56		0.18		0.96		0.18		0.45
< 50%	68	-13% [-15, -8]		-18% [-20, -13]		-9% [-12, -6]		-3% [-3, -2]		2% [1, 3]	
≥ 50%	76	-12% [-17, -7]		-22% [-26, -14]		-8% [-13, -5]		-3% [-4, -2]		1% [-2, 4]	
Lymphovascular space invasion^c^			0.05		**0.004**		0.19		0.07		0.10
No	103	-11% [-14, -6]		-18% [-20, -12]		-8% [-11, -5]		-2% [-3, -1]		2% [1, 3]	
Yes	34	-16% [-23, -11]		-26% [-37, -19]		-9% [-22, -5]		-4% [-6, -2]		1% [-3, 3]	
Histological subtype			0.05		**0.007**		0.09		**0.04**		0.38
EEC grade 1–2	64	-10% [-13, -5]		-14% [-20, -9]		-7% [-10, -5]		-2% [-3, -1]		2% [0, 4]	
EEC grade 3	26	-16% [-20, -5]		-20% [-27, -13]		-9% [-20, 6]		-2% [-4, -1]		1% [-4, 3]	
NEEC	62	-17% [-21, -13]		-25% [-29, -19]		-12% [-17, -7]		-3% [-4, -3]		1% [-1, 3]	
FIGO stage			0.46		0.11		0.75		0.10		0.55
I-II	105	-12% [-15, -10]		-18% [-22, -14]		-9% [-12, -7]		-2% [-3, -1]		1% [0, 3]	
III-IV	47	-16% [-22 -7]		-23% [-29, -18]		-11% [-19, -1]		-4% [-5, -3]		1% [-3 4]	

CI, confidence interval; EEC, endometrioid endometrial carcinoma; FIGO, International Federation of Gynecology and Obstetrics; NEEC, non-endometrioid endometrial carcinoma; SAV, subcutaneous abdominal fat volume; TAV, total abdominal fat volume; VAV, visceral abdominal fat volume; VAV%, visceral-to-total fat percentage; WC, waist circumference

^a^Delta[δ] TAV, VAV, SAV and WC defined as ((follow-up - primary)/primary))

^b^Delta[δ] VAV% defined as (follow-up - primary)

^c^Number (n) of patients with variables missing/not assessed in n_follow−up_: diabetes (1), myometrial invasion (8), lymphovascular space invasion (15)

^d^Lymphadenectomy in 100/152 of the patients in n_follow−up_

*Mann-Whitney U-test for two categories (exact p). Kruskal-Wallis equality-of-populations rank test for three categories. *p* < 0.05 marked in bold

High positive correlations were seen between δTAV, δVAV, and δSAV (*ρ* ≥ 0.70, *p* < 0.001 for all), while δVAV% was only significantly correlated to δVAV (*ρ* = 0.55, *p* < 0.001) (Suppl. Table S4).

#### Temporal changes in CT obesity markers and progression-free survival

Among the 152 patients having a follow-up CT (n_follow−up_), 53 (35%) patients had recurrence/progression diagnosed a median (interquartile range) [range] of 16 (8, 27) [1, 46] months after primary diagnosis. Among these, 44/53 patients had either previously confirmed progression or suspicion of progression at the follow-up CT. The remaining 9/53 patients had no signs of recurrence/progression at follow-up CT, but were diagnosed with recurrence between 1 and 30 months after follow-up CT.

Patients experiencing progression/recurrence exhibited larger reduction in visceral and total fat compartments (δVAV=-24%, δVAV%=-3%, δTAV%=-17%), than patients with no signs of progression/recurrence (δVAV=-17%, δVAV%=-2%, δTAV%=-11%, *p* ≤ 0.03 for all), while no significant difference were observed for δSAV and δWC (*p* ≥ 0.27 for both) (Table 3). Grouping patients in δVAV% quartiles (Fig. 2C-D) showed that patients exhibiting the most pronounced reduction in VAV% (δVAV%<-6%) had significantly lower PFS than patients with δVAV% ≥-6% (Fig. 2D, *p* < 0.01). δVAV%<-6% was also linked to poor survival in univariable Cox analysis (HR [95% CI] = 2.1 [1.2, 3.6], *p* = 0.02), and tended to the same when stratified for FIGO stage I-IV (HR [95% CI] = 1.7 [1.0, 3.0], *p* = 0.08).

Adjuvant treatment regimens for patients with/without progression/recurrence in n_follow−up_ were similar (Suppl. Table S5, *p* = 0.08), and the delta obesity markers were overall similar in patients receiving adjuvant treatment (*n* = 108) and in patients with no adjuvant treatment (*n* = 44, *p* ≥ 0.27 for all). Among patients receiving chemotherapy (102/152 patients in n_follow−up_), patients who developed progression/recurrence (*n* = 38) showed a larger reduction in visceral fat compartments (δVAV=-26%, δVAV%=-4%), than patients who did not develop progression/recurrence (*n* = 64, δVAV=-18%, δVAV%=-2%, *p* ≤ 0.04 for both) (Suppl. Table S6).

## Discussion

This large endometrial cancer study is linking CT assessed visceral adiposity (high VAV% at primary diagnosis) to high-risk histologic features, myometrial invasion, lymphovascular space invasion, and poor outcome. Furthermore, we describe temporal changes in abdominal adiposity markers ∼ 1 year after diagnosis, finding that patients have lower visceral-, subcutaneous-, and total abdominal fat volumes at follow-up compared to primary diagnosis. Importantly, patients experiencing disease progression exhibited larger reductions in visceral adiposity than patient with no progression, suggesting that disproportionally high visceral fat loss during therapy is associated with more aggressive clinical course in endometrial cancer.

Several complex molecular mechanisms explain how obesity may drive tumorigenesis and tumor progression [10, 30, 31]. One hypothesis is that visceral adipose tissue secretes free fatty acids, leading to increased levels of insulin which in itself promotes tumorigenesis [4, 31]. Another mechanism may be that adipose tissue produces cytokines which can lead to a persistent inflammatory environment that further promotes tumorigenesis and poor outcomes [4, 10, 30]. Alterations in steroid hormone metabolism is also thought to play an essential role in endometrial cancer [32]. Patients with endometrial cancer have been shown to have higher blood levels of estradiol than healthy individuals [33]. While VAV is assumed to be a main driver of increased insulin levels and inflammation, its role in production of steroid hormones (e.g. estradiol) and lipids is less investigated. Recently, two endometrial cancer studies reported that estradiol (measured in preoperative blood samples) is positively correlated with both preoperative VAV and SAT (correlation coefficients ranging from 0.54 to 0.74), but not with VAV% [34, 35]. Lipid levels were investigated in one of these studies, but no strong correlations were detected between CT derived obesity markers and the serum lipids investigated (cholesterol, HDL, LDL, NHDL, Triglycerides) [35].

In the present study, we derived CT obesity markers in 293 endometrial cancer patients, diagnosed in 2016–2020. Patients with deep myometrium invasion (MI ≥ 50%), lymphovascular space invasion (LVSI), and high-risk histology (EEC grade 3, NEEC) had significantly higher preoperative VAV% (median values: 35% in MI ≥ 50% and 36% in LVSI, EEC grade 3, and NEEC), than patients with MI < 50%, no LVSI, and low-risk histology (median values: 34% in MI < 50% and no LVSI; 33% in EEC grade 1–2). BMI had an opposite trend, with slightly lower BMI seen in patients with high-risk histology (median values: 27 kg/m^2^ in EEC grade 3 and NEEC) than in patients with low-risk histology (median value: 28 kg/m^2^ in EEC grade 1–2). In a study by Mauland et al. on 227 endometrial cancer patients (diagnosed in 2009–2014, same hospital, but no patient overlap with the present study), similar associations were reported for BMI, with a median BMI of 28 in EECs grade 1–2; 25 in EECs grade 3; and 26 in NEECs (*p* = 0.02) [18]. Mauland et al. did not report significant associations between high VAV% and high-risk histology (*p* = 0.54), and MI and LVSI were not included in their analyses. However, their finding that VAV%≥37% (median value) is associated with poor survival [18], is reproduced in the present study, albeit with a slightly different median VAV% value (VAV% ≥35%) reported in our study. Similar to our findings, no significant correlation between VAV% and BMI was observed by Mauland et al. [18]

Associations between visceral fat volumes and endometrial cancer survival have also been reported for patient subgroups with high-grade histology (EEC grade 3/NEEC) by Donkers et al. (176 patients) [20] or advanced stage (FIGO III-IV) by Buckley et al. (83 patients) [21]. Donkers et al. reported that high VAV% (≥ 34%) predict poor survival in NEEC patients but not in EEC grade 3 patients. Buckley et al. found that FIGO III/IV patients with extreme visceral obesity (VAV/SAV > 45%) had shorter recurrence-free- and overall survival. In the present study, including endometrial cancers of all histological subtypes/grades and FIGO stages, high VAV% (≥ 35%) was significantly linked to poor survival both in the univariable analysis (HR = 2.3) and when stratified for FIGO stage (HR = 3.1). Taking all these findings together, CT assessed VAV% appears to be a relevant prognostic obesity marker in endometrial cancer, clearly outperforming more traditional obesity markers such as weight, waist circumference and BMI. However, since VAV% is associated with other clinical factors (such as patient age and diabetes) and may also be influenced by patient lifestyle (e.g., exercise, diet), its independent role in patient prognosis remains unsettled.

Studies on BMI/weight change patterns in endometrial cancer patients after primary treatment, are inconclusive and show bidirectional effects on survival from weight change during follow-up [36–38]. Matuso et al. conducted a study on 665 endometrial cancer patients and concluded that both weight gain and weight loss were associated with poor survival [36]. In similar studies, Laskov et al. linked weight gain to increased risk of disease recurrence (205 patients) [37], while Santana et al. found no associations between weight change and survival (526 patients) [38]. Visceral adiposity (i.e. high VAV%) at primary diagnosis has been linked by us and others, to poor prognosis in endometrial cancer, and the present study interestingly shows that both VAV and VAV% are decreasing from primary diagnosis to follow-up (δVAV/δVAV%: -20%/-3%, *p* < 0.001 for both) at ∼ 1 year after diagnosis. Moreover, patients who developed disease progression had a larger reduction in visceral adiposity from primary diagnosis to follow-up (δVAV/δVAV%: -24%/-3%), than patients who did not develop progression (δVAV/δVAV%: -17%/-2%, *p* < 0.006 for both). Low δVAV% (≤-6%) was also significantly linked to poor survival both in the univariable analysis (HR = 2.1; *p* = 0.02 and tended to the same when stratified for FIGO stage (HR = 1.7; *p* = 0.08). Previous endometrial cancer studies on BMI/weight change during follow-up are mainly from cohorts where the majority of patients had low FIGO stage and received no adjuvant treatment [36–38]. In our subcohort of patients with follow-up CT scans, 71% (108/152) received adjuvant treatment, consisting of standard chemotherapy in 67% (102/152). Patients undergoing adjuvant chemotherapy will more often develop long-term side-effects and sequela such as neuropathy, lymphedema, fatigue and reduced physical functioning [39]. Furthermore, nausea and lower appetite induced by chemotherapy treatment, could explain a more pronounced weight loss in patients treated with chemotherapy. In our study, no overall differences in the delta obesity markers were found between patients who received/not received adjuvant treatment. Also, among patients receiving chemotherapy (*n* = 102), a larger reduction in visceral adiposity was observed for patients with disease progression (δVAV/δVAV%: -26%/-4%), than for patients with no signs of progression (δVAV/δVAV%: -18%/-2%, *p* ≤ 0.04 for both). Taken together, these findings indicate that the higher visceral fat loss seen in patients with disease progression, is unlikely to be primarily caused by side-effects from more extensive chemotherapy treatments. On the contrary, they are more likely reflecting underlying overall pathophysiology inherently associated with cancer progression, e.g., pain, immobility, altered metabolism and changes in diets due to progressing disease.

The present study is, to date, the largest, population-based endometrial cancer study on abdominal fat distribution, and its role in cancer progression and survival. CT derived visceral obesity markers are easy to obtain from clinically acquired CT scans, and they prove to be robust to interobserver variations, using a predefined HU range and a semi-automated tissue segmentation tool. Describing the changes in abdominal fat distribution from primary diagnosis to follow-ups during and after treatment yields new knowledge, important for a more comprehensive understanding of the role of visceral adiposity in endometrial cancer tumorigenesis and progression. The present study links visceral obesity markers and temporal change in these during treatment, to clinical phenotype, histological features, and patient outcome in endometrial cancer. Combining these markers, with other relevant clinical-, imaging- and histological biomarkers, could assist in tailoring treatment and predicting outcomes in endometrial cancer. However, this is the first endometrial cancer study describing changes in abdominal fat distribution patterns during treatment, and the findings should be validated in independent larger patient cohorts. Furthermore, the underlying mechanisms driving the changes in the fat distribution during and after cancer treatment need to be further investigated.

One limitation of the study is that the follow-up cohort is enriched for higher grade- and stage disease, compared with the cohort at primary diagnosis. Follow-up of patients with low-risk disease and no signs of recurrence/progression are mostly performed at local hospitals/gynecologists and the use of CT as part of the follow-up is variable in this patient group. The delta obesity measures reported in the present study, do hence represent longitudinal obesity changes in a selected high-risk follow-up cohort in whom recurrence is more likely, and are thus not necessarily representative for all endometrial cancer patients.

A second limitation is that while most of the follow-up CTs (93%) in this study are performed as diagnostic scans with CT contrast agent, only 23% of the preoperative CT examinations were contrast-enhanced. CT contrast agent will inherently influence Hounsfield units, especially in tissues with large contrast enhancement. Different scanner technologies, protocol settings, patient anatomy and image artifacts can also have an impact on image quality, and hence the semi-automatic fat segmentation. However, all images were inspected visually, and the segmentations were manually corrected if deemed necessary. During these visual inspections we did not detect any large effects of CT contrast or image quality on the abdominal fat tissues examined. It is thus highly unlikely that these issues have largely influenced the accuracy of the segmented adipose tissue volumes.

The biological relationship between obesity and cancer is complex, involving several potential mechanisms such as alterations in insulin metabolism, inflammatory responses and steroid metabolism. This retrospective study did only assess CT obesity markers in relation to routinely acquired clinicopathological features and outcomes, without incorporating extensive data on insulin levels, inflammatory signaling or hormonal data, which could be of interest to understand their relation to the derived CT obesity markers. In addition, molecular profiles, recently incorporated in the revised FIGO 2023 staging system, were not available for the patients in this retrospective study, but this information would be valuable to incorporate in future studies.

## Conclusion

This large endometrial cancer study shows that CT derived visceral obesity (increased visceral-to-total fat percentage) is associated with high-risk histologic features, myometrial and lymphovascular space invasion and poor prognosis. Furthermore, patients experiencing disease progression have a more pronounced reduction in visceral fat volumes at follow-up. Our findings confirm that visceral fat percentage can be a useful tool for prediction of outcomes in endometrial cancer. However, future studies are needed to identify the underlying biological mechanisms that links visceral adiposity to tumorigenesis, progression and survival in endometrial cancer.

## Electronic supplementary material

Below is the link to the electronic supplementary material.

Supplementary Material 1

## Data Availability

The data sets generated and analyzed during the current study are not publicly available but are available from the corresponding author upon reasonable request, and if in compliance with the general data protection regulation (GDPR) and patient consents.

## Abbreviations

BMI: Body Mass Index
EEC: Endometrioid Endometrial Carcinoma
NEEC: Non-Endometrioid Endometrial Carcinoma
FIGO: International Federation of Gynecology and Obstetrics
LNM: Lymph Node Metastases
SAV: Subcutaneous Abdominal fat Volume
TAV: Total Abdominal fat Volume
VAV: Visceral Abdominal fat Volume
VAV%: Visceral-to-total abdominal fat percentage
WC: Waist Circumference
