# Supplementary material for: Abdominal fat distribution in endometrial cancer: from diagnosis to follow-up
Source: BMC Cancer. 2025 May 15;25:879. doi: 10.1186/s12885-025-14155-3 (PMC12079972; doi:10.1186/s12885-025-14155-3)
Supplement: Supplementary file 1 — Supplementary Material 1 [file 12885_2025_14155_MOESM1_ESM.docx]

# Supplementary material

| **Supplementary Table S1**  Intraclass correlation coefficients (ICC) with 95% confidence intervals (CI) for the CT abdominal obesity markers of 21 patients based on mean-rating of two independent readers (ML and JS), with absolute-agreement, two-way random-effects model | | | | | |
| --- | --- | --- | --- | --- | --- |
|  | **TAV**  [95% CI] | **VAV**  [95% CI] | **SAV**  [95% CI] | **VAV%**  [95% CI] | **WC**  [95% CI] |
| Average ICC | 0.998  [0.996, 0.999] | 0.998  [0.964, 1.000] | 0.997  [0.990, 0.999] | 0.938  [0.821, 0.976] | 0.998  [0.990, 0.999] |
| p* | <0.001 | <0.001 | <0.001 | <0.001 | <0.001 |
| SAV, subcutaneous abdominal fat volume; TAV, total abdominal fat volume; VAV, visceral abdominal fat volume; VAV%, visceral-to-total fat percentage; WC, waist circumference  *F test that ICC=0.00 | | | | | |

| **Supplementary Table S2**  Correlations coefficients (Spearman’s rho (ρ)) between body mass index (BMI), computed tomography (CT) abdominal obesity markers, and age at primary diagnosis in n_primary_=293 endometrial cancer patients | | | | | | | |
| --- | --- | --- | --- | --- | --- | --- | --- |
|  | **Median [range]** | **BMI^a^ (ρ)** | **TAV (ρ)** | **VAV (ρ)** | **SAV (ρ)** | **VAV% (ρ)** | **WC (ρ)** |
| BMI^a^ | 28 [17, 59] kg/m^2^ | - | - | - | - | - | - |
| TAV | 8811 [1547, 32730] ml | 0.90* | - | - | - | - | - |
| VAV | 3227 [264, 9863] ml | 0.78* | 0.91* | - | - | - | - |
| SAV | 5660 [993, 25617] ml | 0.90* | 0.97* | 0.79* | - | - | - |
| VAV% | 35 [13, 57] % | -0.12 | -0.02 | 0.35* | -0.22* | - | - |
| WC | 98 [63, 147] cm | 0.89* | 0.93* | 0.86* | 0.90* | -0.02 | - |
| Age^b^ | 69 [27, 90] years | -0.08 | -0.09 | 0.06 | -0.17** | 0.41* | -0.04 |
| SAV, subcutaneous abdominal fat volume; TAV, total abdominal fat volume; VAV, visceral abdominal fat volume; VAV%, visceral-to-total fat percentage; WC, waist circumference  ^a^BMI missing for n=8 patients  ^b^Age at primary diagnosis  *Spearman’s ρ, p<0.001  ** Spearman’s ρ, 0.001<p<0.05 | | | | | | | |

| **Supplementary Table S3**  Univariable progression free survival Cox proportional hazard ratios’ (HRs) for each of the computed tomography derived obesity markers and body mass index (BMI), dichotomized at median. VAV% depicted significant HR in univariable analyses and were further stratified for FIGO stage I-IV, including an interaction term between VAV% and age. All analyses were performed on variables derived at primary diagnoses (n_primary_=293) | | | | |
| --- | --- | --- | --- | --- |
| **Dichotomized variable** | **Univariable HR [95% CI]** | **p*** | **Stratified** HR [95% CI]** | **p**** |
| TAV | 0.8 [0.5, 1.3] | 0.36 | - | - |
| VAV | 1.1 [0.7, 1.9] | 0.59 | - | - |
| SAV | 0.7 [0.5, 1.2] | 0.24 | - | - |
| VAV% | 2.3 [1.1, 5.1] | **0.03** | 3.1 [1.4, 6.9] | **0.005** |
| WC | 0.9 [0.6, 1.5] | 0.78 | - | - |
| BMI | 0.8 [0.5, 1.4] | 0.50 | - | - |
| CI, confidence interval; HR, hazard ration; SAV, subcutaneous abdominal fat volume; TAV, total abdominal fat volume; VAV, visceral abdominal fat volume; VAV%, visceral-to-total fat percentage; WC, waist circumference  *Cox proportonial Hazard Regression model, including an interaction term between VAV% and age </≥69 years, p<0.05 marked in bold  **Cox proportonial Hazard Regression model including an interaction term between VAV% and age </≥69 years, and stratified for FIGO stage I-IV, p<0.05 marked in bold | | | | |

| **Supplementary Table S4**  Correlations coefficients (Spearman’s rho (ρ)) between delta [δ] computed tomography (CT)-derived abdominal obesity markers from primary diagnosis to CT follow-up, and age at primary diagnosis in n_follow-up_=152 endometrial cancer patients | | | | | |
| --- | --- | --- | --- | --- | --- |
|  | δ**TAV (ρ)** | δ**VAV (ρ)** | δ**SAV (ρ)** | δ**VAV% (ρ)** | δ**WC (ρ)** |
| δVAV | 0.85* | - | - | - | - |
| δSAV | 0.96* | 0.70* | - | - | - |
| δVAV% | 0.12 | 0.55* | -0.10 | - | - |
| δWC | 0.54* | 0.42* | 0.56* | -0.05 | - |
| Age | -0.33* | -0.29 | -0.29* | -0.12 | -0.33* |
| SAV, subcutaneous abdominal fat volume; TAV, total abdominal fat volume; VAV, visceral abdominal fat volume; VAV%, visceral-to-total fat percentage; WC, waist circumference  *Spearman’s ρ, p<0.001 | | | | | |

| **Supplementary Table S5**  Adjuvant treatment for patients with follow-up CT (n_follow-up_=152) | | | |
| --- | --- | --- | --- |
|  | **Progression/recurrence** | | |
|  | **Yes (n=53)**  n (%) | **No (n=99)**  n (%) | **p*** |
| Chemotherapy | 38 (71%) | 64 (65%) | 0.08 |
| External/internal radiation | 2 (4%) | 2 (2%) |  |
| Hormonal treatment | 1 (2%) | 0 (0%) |  |
| Chemoradiation | 1 (2%) | 0 (0%) |  |
| No adjuvant treatment | 11 (21%) | 33 (33%) |  |
| *Mann-Whitney U test exact p | | | |

| **Supplementary Table S6**  Change (delta [δ], %) in computed tomography (CT)-derived abdominal obesity markers from primary diagnosis to CT follow-up scans in patients receiving adjuvant chemotherapy (n=102). The delta obesity markers are given separately for patients with progression/recurrence (n=38), and patients with no progression/recurrence during follow-up (n=64). The CT follow-up scan were acquired in a median (interquartile range) of 13 (7, 19) months after primary diagnosis, The follow-up time were 49 (38, 61) [median (interquartile range)] months for patients with no progression/recurrence. For patients with progression/recurrence the time from primary diagnosis to progression/recurrence were 18 (8, 28) [median (interquartile range)] months | | | |
| --- | --- | --- | --- |
|  | **Progression/recurrence** | | |
|  | **Yes** (n=38)  Median [95% CI] | **No** (n=64)  Median [95% CI] | **p*** |
| δTAV | -17% [-29, -9] | -12% [-17, -7] | 0.11 |
| δVAV | -26% [-37, -18] | -18% [-23, -13] | **0.02** |
| δSAV | -13% [-27, 3] | -8% [-12, -6] | 0.27 |
| δVAV% | -4% [-6, -3] | -2% [-3, -1] | **0.04** |
| δWC | 1% [-4, 4] | 1% [-1, 3] | 0.27 |
| CI, confidence interval; δ, delta ((follow-up – primary)/primary); SAV, subcutaneous abdominal fat volume; TAV, total abdominal fat volume; VAV, visceral abdominal fat volume; VAV%. visceral-to-total fat percentage; WC, waist circumference  *Mann-Whitney U test exact p (p<0.05 marked in bold) | | | |
